# Heimann-Bielschowsky phenomenon and hypotropic DVD in case of monocular vision loss: a case report

**DOI:** 10.1186/s12886-020-01502-0

**Published:** 2020-06-26

**Authors:** Hyung Jun Choi, Bo Young Chun

**Affiliations:** 1 Department of Ophthalmology, School of Medicine, Kyungpook National University, Daegu, South Korea; 2 Brain Science & Engineering Institute, School of Medicine, Kyungpook National University, 680 Gukchaebosang Street, Jung-gu, Daegu, 700-422 South Korea

**Keywords:** Heimann-Bielschowsky phenomenon, Hypotropic DVD, Monocular vision loss, Case report

## Abstract

**Background:**

The Heimann-Bielschowsky phenomenon (HBP) is an underrecognized condition characterized by slow, pendular, vertical oscillations of the eye accompanying monocular vision loss. Hypotropic dissociated vertical deviation (DVD) is another rare condition induced by asymmetric visual input. This report documents a rare case of HBP with hypotropic DVD.

**Case presentation:**

This report describes a case of a 58-year-old woman with HBP and hypotropic DVD, having suffered monocular vision loss in the left eye due to blunt trauma at the age of 10. Preoperatively, she was orthophoric at near fixation and demonstrated an intermittent, slow hypotropia of the left eye upon distance fixation that never rose above the midline. She underwent a 7 mm recession of the inferior rectus muscle in the left eye. After surgery, intermittent, downward drifts became constant vertical oscillations at both distance and near fixation.

**Conclusions:**

This case describes the clinical manifestation of an eye movement disorder related to prolonged monocular vision loss.

## Background

The Heimann-Bielschowsky phenomenon (HBP) is characterized by slow, pendular, vertical oscillations of equal velocity in both directions [1, 2] and typically occurs in an eye with severe vision loss. The condition remains underrecognized and is not discussed in most standard strabismus textbooks [1, 3]. Hypotropic dissociated vertical deviations (DVD) are also associated with asymmetric visual input and defined as a dissociated hypodeviation of one eye which occurs intermittently while the other eye is fixating a distant target [4]. To the best of our knowledge, a case presenting with both HBP and hypotropic DVD has yet to be reported. Herein, we report a rare case of vertical oscillations in monocular vision loss manifesting with clinical characteristics of HBP and hypotropic DVD.

## Case presentation

A 58-year-old female presented with a 4-year history of slow, intermittent, downward drift in her left eye. Blunt trauma to the left eye at 10 years of age left her with severe vision impairment therein. About 35 years later, she was diagnosed with traumatic cataract and sensory esotropia in the left eye and underwent left cataract extraction and recession of the medial rectus muscle, and resection of the lateral rectus muscle. Slow, intermittent, downward drifting of the left eye subsequently developed upon distance fixation and increased steadily in size and frequency over the next 4 years. The patient did not experience diplopia due to her poor vision of the left eye. Her visual acuities were 20/20 in her right eye and 20/400 in her left eye without glasses and 20/200 with glasses (+ 1.75 Dsph = − 3.0 Dacylax 180); however, the patient refused to wear glasses or contact lenses after cataract surgery.

**Fig. 1:**
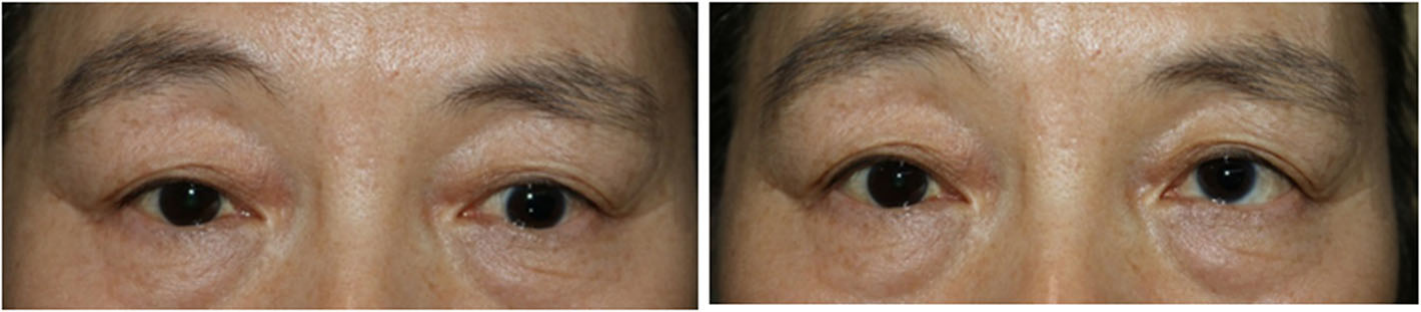
Left) Preoperative photograph demonstrating hypotropic DVD of the left eye as the right eye fixates on a distant target. Right) Preoperative photograph demonstrating nearly orthophoric as the left eye fixates on a near target


Eye movements were normal, and no oblique muscle dysfunction was found. When the patient fixated at a distance, she demonstrated an intermittent, slow, downward drift of the left eye (approximately 0.5 Hz frequency) that never rose above the midline, of variable magnitude ranging up to 30 prism diopters in the primary position. (Fig. 1; Additional file 1) She reported that a slow, downward drift occurred during fatigue, inattention, or distance viewing. When she attempted to fixate with her left eye at a near target, she was orthophoric with no vertical movement in either eye. (Fig. 1) The Bielschowsky head-tilt test demonstrated no significant difference between primary and head-tilt positions. She had a left relative afferent pupillary defect. The anterior and posterior segments were healthy with posterior chamber lens implant of the left eye. She had no other neurologic disorders. A brain magnetic resonance imaging failed to reveal any abnormalities of extraocular muscles or pulley. Laboratory work-up to rule out ocular myasthenia and thyroid eye disease was negative for both.

**Additional file 1.** Preoperative video demonstrating an intermittent, slow, downward drift of the left eye that never rose above the midline, of variable magnitude ranging up to 30 prism diopters in the primary position.


**Fig. 2:**
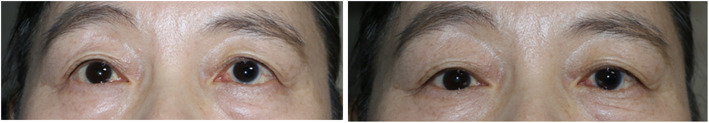
Left) Postoperative photograph demonstrating slight hypertropia of the left eye as the right eye fixates on a distant target. Right) Postoperative photography demonstrating orthophoric alignment as the left eye fixates on a near target


The patient was diagnosed with hypotropic DVD in the left eye. To correct her hypotropia at distance, she underwent a 7 mm recession of the inferior rectus muscle in her left eye. At 3 months after surgery, she was orthophoric at near fixation and slightly hypertropic to 8 prism diopters at distance fixation (Fig. 2). Left eye downward drifts were slightly reduced in vertical amplitude but not frequency. In addition, intermittent downward drifts occurring at distance fixation became constant vertical oscillations that crossed the midline after surgery. When she attempted to fixate with her left eye at a near target, she was orthophoric for about 10 s, followed by slow, pendular oscillations of the left eye (Additional file 2). Her ocular manifestations before and after strabismus surgery suggested clinical characteristics of both hypotropic DVD and HBP.

**Additional file 2.** Postoperative video demonstrating slow, pendular oscillations of the left eye following orthophoric potision when she attempted to fixate with her left eye at a near target.


## Discussion and conclusions

HBP is an underrecognized condition characterized by slow, pendular, vertical oscillations of equal velocity in both directions [1, 2]. The mechanism of the HBP remains unknown, although it has been largely attributed to disruption of the fusional vergence mechanism or monocular visual stabilization system following profound vision loss in one eye [1, 3]. Hypotropic DVD is also associated with asymmetric visual input and defined as a dissociated hypodeviation of one eye which develops intermittently while the other eye is fixated on a distant target [4]. The etiology of hypotropic DVD is also unknown. Lim [4] postulated that asymmetric visual input between the two eyes might disrupt the vertical vergence system, inducing an abnormal vertical divergence movement that could induce hypotropic DVD in certain patients. Although HBP and hypotropic DVD share similar etiologies related to monocular vision loss, each has different clinical manifestations. Patients with HBP demonstrate vertical eye oscillation from below the horizontal to above the midline; however, intermittent downward drifts of the eye never rise above the midline in patients with hypotropic DVD [4–6]. In addition, HBP movements have been characterized by pendular vertical oscillations, while the eye movements in hypotropic DVD are not nystagmoid but more sporadic or intermittent, only occurring upon distance fixation [4–6].

This report describes a case of HBP with hypotropic DVD in an eye with monocular vision loss. Prior to strabismus surgery, the patient demonstrated distance/near fixation disparity, that is, orthophoric at near fixation and intermittently hypotropic at distance fixation, a typical presentation of hypotropic DVD [4–6]. The patient used the normal vision right eye exclusively for distance fixation, leading to intermittent downward drifts of the poor vision left eye. At near fixation, the patient attempted to recruit the left eye to achieve peripheral fusion and was orthophoric at near fixation. Preoperative, intermittent downward drifts of the left eye remained below the horizontal meridian, another typical manifestation of hypotropic DVD [4–6]. Hypotropic DVD has some striking similarities with hypertropic DVD including slow, vertical drift which returns back to the midline position, the dissociated nature of the deviation, intermittent deviation depending on the fusional status, and potential association with horizontal strabismus [4]. However, there are a few notable differences between hypotropic and hypertropic DVD. First, hypotropic DVD demonstrates downward drifts of the deviating eye without any torsional eye movements; second, hypotropic DVD is not related to latent nystagmus; third, hypotropic DVD is mostly acquired and is not associated with congenital esotropia; finally, contrary to hypertropic DVD, hypotropic DVD can be overcorrected by surgery [4].

After strabismus surgery, the two cardinal manifestations of hypotropic DVD disappeared, probably due to the large recession of the inferior rectus muscle in her left eye. However, intermittent downward drifts in the left eye only observed at distant fixation became constant, pendular, vertical oscillations in the left eye at both near and distant fixation. We assume that the strabismus surgery led to further disruption of central fusion and the fusional vergence system in addition to the preexisting abnormal vertical vergence system caused by prolonged monocular vision loss. However, prior to strabismus surgery, these two conditions could have coexisted. Large recession of the inferior rectus muscle successfully corrected the hypotropic DVD, after which HBP became the dominant manifestation in the eye with monocular vision loss.

To the best of our knowledge, this is the first report of HBP with hypotropic DVD. The etiologies of HBP and hypotropic DVD are both related to long-standing, profound vision loss in one eye, suggesting that one or both could develop in eyes with prolonged vision loss.

## Data Availability

The data generated during the present study is available upon request from the corresponding author.

## Abbreviations

DVD: Dissociated vertical deviations
HBP: Heimann-Bielschowsky phenomenon
